# A limitation of Chase’s proposal for safe doubling of ventilator capacity

**DOI:** 10.1186/s13054-020-03237-2

**Published:** 2020-08-18

**Authors:** Ross Freebairn, Michael Park

**Affiliations:** 1 Hawke’s Bay Hospital, Omahu Road, Hastings, 4221 New Zealand; 2 University of Otago, Dunedin, New Zealand

Letter:

We read with interest the description by Chase and colleagues of a system to provide an “in series” rather than “in parallel” sharing of a single ventilator between two patients [1]. Their suggestion is that this is a “last resort technique” that may be useful during the COVID-19 pandemic surge. In contradistinction to other “shared ventilation” systems, Chase’s design permits individual adjustments for patients’ compliance and PEEP [2]. However, using the system described by Chase, every second breath is delivered to each patient. This results in an inspiration time limited to less than 50% of the respiratory cycle time, even if the ventilator set expiration time is minimized [3]. Several ventilators further limit inspiratory time to 80% of cycle time, meaning the longest inspiratory time would be an I:E ratio of 1:1.5 [4].

COVID-19 respiratory failure is characterized by severe hypoxemia, often with near normal compliance, and frequently resistant to PEEP and recruitment maneuvers [5]. One additional strategy available to increase oxygenation is to increase inspiratory time, which allows more distribution of fresh gas to slow filling alveoli and raises the mean alveolar pressure. The Chase apparatus’s inability to provide reverse ratio or even 1:1 ventilation is an important limitation in severe hypoxemia respiratory failure that may reduce its utility in a COVID-19 crisis.

## Data Availability

Not applicable

## References

[1] Chase JG, Chiew YS, Lambermont B, Morimont P, Shaw GM, Desaive T (2020). Safe doubling of ventilator capacity: a last resort proposal for last resorts. Crit Care.

[2] Epstein D, Hoffman Y, Dahoud G, Raz A, Miller A. Simultaneous ventilation of two simulated ARDS patients in COVID-19 pandemic. Critical Care. 2020;24(1):214. 10.1186/s13054-020-02940-4 PMC7213771 32393390

[3] Puritan Bennett 840 Ventilator [technical data ]. [Available from: https://www.medtronic.com/content/dam/covidien/library/us/en/product/acute-care-ventilation/puritan-bennett-980-ventilator-system-tech-specifications.pdf. Accessed 24 July 2020.

[4] Hamilton Medical C6 Ventilator [technical data]. [Available from: https://www.hamilton-medical.com/dam/jcr:0d8ebdcc-55d5-4604-86cc-e98d3ade7ead/HAMILTON-C6-tech-specs-en-689596.02.pdf. Accessed 24 July 2020.

[5] Gattinoni L, Chiumello D, Rossi S (2020). COVID-19 pneumonia: ARDS or not?. Crit Care.

